# A Diagnostic Dilemma: Transposition of the Great Arteries

**DOI:** 10.7759/cureus.38931

**Published:** 2023-05-12

**Authors:** Victor N Oboli, Anthony Pizzolla, Priyam Pattnaik

**Affiliations:** 1 Pediatrics, New York City Health + Hospitals/Lincoln, New York, USA; 2 Pediatrics, St. George’s University School of Medicine, True Blue, GRD; 3 Neonatology, New York City Health + Hospitals/Lincoln, New York, USA

**Keywords:** arterial switch surgery, prenatal ultrasonography, duct-dependent heart defects, cyanotic congenital heart disease, transposition of the great arteries

## Abstract

Transposition of the great arteries (TGA) remains one of the most common and severe underdiagnosed congenital cardiac anomalies in the prenatal period. Unfortunately, despite advances in prenatal ultrasound screening, the detection rate of major congenital heart defects (CHDs) remains low. We present the case of a preterm male infant delivered limp with generalized cyanosis and in respiratory distress at 36 weeks gestation with postnatal echocardiography (ECHO) depicting dextro-TGA (d-TGA). Maternal prenatal targeted fetal anomaly ultrasonography at 18 weeks gestation showed abnormal right ventricle and right ventricular outflow tract. Subsequent two-time repeat fetal ECHO showed ventricular septal defect. This case represents how challenging and unrecognized critical CHDs can be. Furthermore, it highlights the need for clinicians to have a high index of suspicion when newborns present with clinical manifestations of critical CHDs and manage it accordingly to avoid severe complications.

## Introduction

Transposition of the great arteries (TGA) is the most common cyanotic heart defect, and approximately 1,153 babies with TGA are delivered annually in the United States [1]. It makes up to 7% of all congenital heart defects (CHDs) [2-4]. Despite advances in prenatal ultrasound screening, the detection rate of major CHDs remains low. Recent reports demonstrate how TGA can be missed in the prenatal period using fetal echocardiography and has less than 50% detection rates [5]. However, over the past years, its detection and diagnosis rates have improved prenatally with advanced three-dimensional (3D), four-dimensional (4D), and spatiotemporal image correlation technology [6].

TGA has two types, namely, dextro-TGA (d-TGA) and levo-TGA (l-TGA). In d-TGA, the aortic valve is positioned in front and on the right side of the pulmonary valve. In contrast, in l-TGA, the aortic valve is located at the left of the pulmonary valve. Additionally, TGA is classified as either simple (isolated) or complex. Simple TGA has no associated cardiac anomalies and is more common, while complex TGA coexists with other cardiac malformations [7]. Common cardiac defects associated with TGA include ventricular septal defect (VSD) and left/right ventricular outflow obstructions. Delays in diagnosing a critical CHD, especially a cyanotic CHD such as TGA, can lead to rapid hemodynamic decompensation or compromise, significant morbidity, and death of a newborn. The essence of prenatal detection of CHD is to improve perinatal outcomes and optimize successful transition in the newborn period [8].

## Case presentation

We present a late preterm male neonate delivered at 36 weeks gestational age (GA) by vaginal delivery to a mother with no significant medical history but inconsistent prenatal care and preterm premature rupture of the membranes. The maternal antenatal history was significant for a targeted fetal anatomy ultrasound scan at 18 weeks GA depicting abnormal right ventricle (RV) and right ventricular outflow tract (RVOT). Subsequent two-time repeat fetal echocardiography (ECHO) showed VAS, and the plan was critical CHD screening, limb blood pressures, and repeat neonatal ECHO after birth.

**Figure 1:**
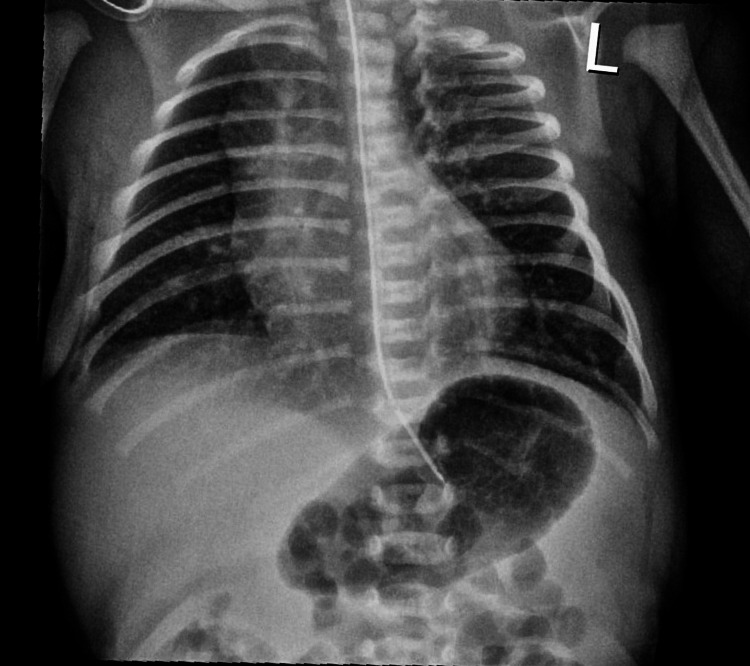
Portable frontal chest radiograph showing normal mediastinum, cardiothymic silhouette, and symmetric lung volumes. No diaphragmatic hernia seen.

**Figure 2:**
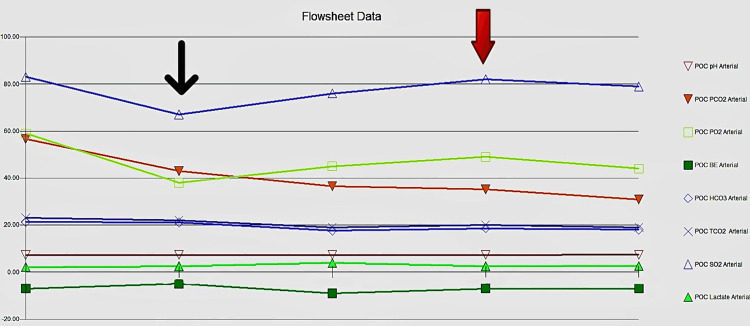
Flowsheet data of the serial arterial blood gas performed. The thin black arrow pointing downward depicts the first arterial blood gas done on admission showing metabolic and respiratory acidosis. The red arrow shows the improvement in acidosis following prostaglandin therapy. POC = point-of-care; PCO_2_ = partial pressure of carbon dioxide; PO_2_ = partial pressure of oxygen; BE = base excess; HCO_3_ = bicarbonate; TCO_2_ = carbon dioxide test

The baby was delivered limp and unresponsive, but the heart rate (HR) was above 100 beats/minute. The baby had generalized cyanosis with poor respiratory effort requiring positive pressure ventilation (PPV) via bag-mask ventilation (BMV) per the Neonatal Resuscitation Program protocol. On physical examination, he had a grade III/VI systolic murmur on the third left intercostal space with no dysmorphic features present. His birth weight was 2,450 g, and APGAR scores were 5, 7, and 7 at one, five, and ten minutes, respectively. PPV with BMV was done for approximately two minutes with FiO_2_ of 50% with an improvement of grimace, respiratory effort, and crying. However, the baby remained cyanotic and in respiratory distress with preductal oxygen of 70% at three minutes of life. PPV was changed to continuous positive airway pressure with 100% FiO_2_. However, he remained centrally cyanotic with oxygen saturations in the late 70s and HR in the 140s. He was admitted to the neonatal intensive unit (NICU) for further evaluation of possible hypoxic respiratory failure, persistent pulmonary hypertension of the newborn, and CHD. His vital signs on NICU admission were temperature - 98.6°F, respiratory rate (RR) - 68/minute, right leg blood pressure (BP) - 75/52 mmHg, and preductal and postductal oxygen saturations in the early 70s and 80s, respectively. Due to persistent low oxygen saturations, he was intubated and started on mechanical ventilation with 100% FiO_2_ which did not improve the preductal and postductal saturations. For suspicion of surfactant deficiency, surfactant was administered to improve oxygenation. Chest X-ray was normal (Figure 1). Arterial blood gas showed respiratory and metabolic acidosis (Figure 2), and random blood glucose was 77 mg/dL. He received three saline boluses due to metabolic acidosis. An umbilical line was placed, and he was started on parenteral nutrition. Sepsis protocol was initiated, and broad-spectrum antibiotics were started.

**Figure 3:**
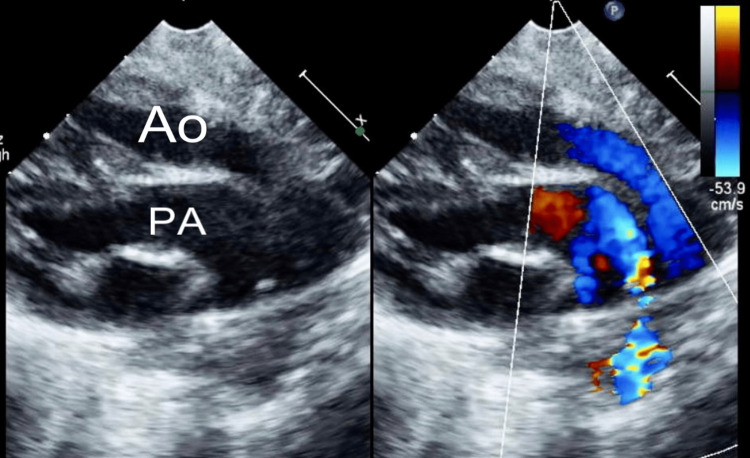
A two-dimensional Doppler echocardiogram with color flow demonstrating the great vessels with the rightward aorta (Ao) located anteriorly to the posterior pulmonary artery (PA) depicting transposition of the great arteries.

With the persistence of more than a 10-point difference between the preductal and postductal oxygen saturations, preductal being lower in the low 70s, he was started on intravenous prostaglandin therapy after discussing with the on-call cardiologist. An emergent repeat ECHO depicted dextro-malposition of the great arteries (Figure 3), intact ventricular septum, large patent ductus arteriosus (PDA), restrictive atrial septal defect (ASD), mild tricuspid and mitral valve regurgitation, and severely dilated left atrium**.** He was transferred to a cardiac facility where he underwent balloon atrial septostomy and later had an arterial switch procedure with ASD closure and PDA ligation. He tolerated the surgery very well and received furosemide for two months post-surgery, and he continues to follow-up in the cardiology outpatient clinic without any concerns. 

## Discussion

D-TGA is a medical emergency that presents with cyanosis and severe hypoxemia after birth. During intrauterine fetal life, the fetomaternal circulation ensures optimal oxygenation and nutrients to the developing fetus. However, hypoxemia ensues in the immediate postnatal life when the ductus arteriosus begins to close with a minimal mixture of pulmonary and systemic circulation through the PDA. In TGA, there is a switch in the embryological origins of the aorta and pulmonary artery from the truncus arteriosus, leading to hypoxemia. If the patency of the PDA is not maintained by administering a prostaglandin E1 (PGE1) analog, severe hypoxemia ensues. D-TGA manifests with central cyanosis, tachypnea, and moderate-to-severe hypoxemia within the first hours or days of life. Hypoxemia depends on the level and degree of atrial blood shunting and patency of the ductus arteriosus. Therefore, early diagnosis and intervention with PGE1 in the immediate postnatal life are paramount to preventing prolonged hypoxemia, acidosis, and death [8].

Prenatal detection of TGA varies widely, and therefore, it is vital to consider various factors responsible for this variation in case it is missed in utero. These factors include examiner experience, GA, ultrasound transducer, cardiac views, and imaging parameters [6]. Hence, neonatologists and emergency physicians should have a high index of suspicion during the immediate postnatal period when newborns are delivered unresponsive with central cyanosis and have clinical features of critical CHD like our patient had. To improve the sensitivity of CHD ultrasound screening, the three-vessel tracheal (3VT) view during fetal ECHO was added to the four-chamber cardiac view in the cardiac ultrasound screening protocol. It involves evaluating the aorta, pulmonary artery, and superior vena cava. This 3VT view defines the relationship between the trachea and the three vessels (3V). The advantage of the 3VT is that an abnormal 3V view most likely points to specific abnormalities such as TGA, tetralogy of Fallot, and VSD that would typically appear normal on a four-chamber view [6]. According to the American Institute of Ultrasound in Medicine and the American College of Radiology, the ultrasonic view allows the assessment of all chambers, and the four-chamber view is mainly utilized. However, an ultrasonic view does not give enough room to visualize the outflow tracts. Therefore, a base view superior to the four-chamber view is more beneficial when evaluating the crossing of the great vessels in utero. A combination of both the base view and the four-chamber view increases the sensitivity and accuracy of diagnosing fetal cardiac anomalies in utero [9]. Although cardiac screening examination is performed optimally between 18 and 22 weeks GA, a systematic review analyzing the accuracy of diagnosing fetal cardiac anomalies, including TGA at 11-14 weeks gestation, found that more than half of fetuses affected by a significant cardiac pathology such as TGA were identified in the first-trimester ultrasound examination [6,10].

Another important consideration, according to Al-Fahham et al., is the quality of images obtained during the second-trimester standard anomaly scan (SAS), which could play a role in missing these diagnoses [11]. A case-control study found that as opposed to factors such as fetal positioning and maternal body mass index, inadequate adaptational skills when performing the SAS was a more important factor contributing to the missed finding. In cases that went undetected, the quality of the cardiac views was inadequate compared to those detected. To this effect, about 31% of cases were unrecognized despite good-quality images [12]. 3D and 4D ultrasonography with spatiotemporal image correlation (4D-STIC) allows obtaining fetal cardiac volumes and their static and real-time analysis in multiplanar and rendering modes. 4D-STIC is a more robust solution in diagnosing and predicting fetuses with TGA [13].

Additionally, Rizzo et al. also demonstrated that using a sonographic-based automated volume count enables the 4D-STIC and facilitates the identification of these cardiac anomalies, mostly TGAs [14]. Irrespective of these available imaging techniques and advancements in diagnostic technologies, CHDs are still continually being missed. Despite the application and advantage of 3D and 4D in diagnosing CHD, these technologies are only available in some facilities during routine fetal cardiac screening, further contributing to the number of missed or unrecognized diagnoses [15,16].

In the immediate postnatal life, the management of TGA involves obtaining immediate echocardiography, which is diagnostic and depicts switched or transposed ventricular-arterial connections. Administration of PGE1 to improve oxygenation should be completed on time. Respiratory support with serial arterial blood gas measurement, prompt acidosis correction, and euglycemia maintenance should also be instituted. Rashkind balloon atrial septostomy is performed for infants who remain hypoxic despite PGE1 administration or have contraindications to immediate definitive surgery. The standard treatment for TGA is cardiac surgery which involves arterial switch procedures, depending on the type of TGA involved [8]. Contemporary studies indicate that surgical correction of TGA results in favorable outcomes and high survival rates. In addition, patients who undergo this procedure can expect to live into adulthood without experiencing any significant health issues [17].

## Conclusions

TGA is a medical emergency and poses an immediate threat to the newborn in the immediate postnatal life. Early diagnosis and intervention in the immediate postnatal life are paramount to preventing prolonged hypoxemia, acidosis, and death before the definitive surgical correction. Regardless of the missed and unrecognized prenatal diagnosis of TGA and other CHDs, clinicians should have a high index of suspicion when newborns present with clinical manifestations of critical CHD and manage it accordingly to avoid drastic complications.

## References

[1] (2023). Congenital heart defects - dextro-transposition of the great arteries (d-TGA). https://www.cdc.gov/ncbddd/heartdefects/d-tga.html.

[2] Garne E, Loane M, Dolk H (2005). Prenatal diagnosis of severe structural congenital malformations in Europe. Ultrasound Obstet Gynecol.

[3] Hoffman JI, Kaplan S (2002). The incidence of congenital heart disease. J Am Coll Cardiol.

[4] Unolt M, Putotto C, Silvestri LM (2013). Transposition of great arteries: new insights into the pathogenesis. Front Pediatr.

[5] Gardiner HM, Kovacevic A, van der Heijden LB (2014). Prenatal screening for major congenital heart disease: assessing performance by combining national cardiac audit with maternity data. Heart.

[6] International Society of Ultrasound in Obstetrics and Gynecology, Carvalho JS, Allan LD (2013). ISUOG Practice Guidelines (updated): sonographic screening examination of the fetal heart. Ultrasound Obstet Gynecol.

[7] Paladini D, Rustico M, Todros T (1996). Conotruncal anomalies in prenatal life. Ultrasound Obstet Gynecol.

[8] Kliegman R, Stanton B, St Geme JW, Schor NF, Behrman RE, Nelson WE (2020). Nelson Textbook of Pediatrics.

[9] Barboza JM, Dajani NK, Glenn LG, Angtuaco TL (2002). Prenatal diagnosis of congenital cardiac anomalies: a practical approach using two basic views. Radiographics.

[10] Karim JN, Bradburn E, Roberts N, Papageorghiou AT (2022). First-trimester ultrasound detection of fetal heart anomalies: systematic review and meta-analysis. Ultrasound Obstet Gynecol.

[11] Al-Fahham MM, Gad NA, Ramy AR, Habeeb NM (2021). Clinical utility of fetal echocardiography: an Egyptian center experience. Egypt Heart J.

[12] van Nisselrooij AE, Teunissen AK, Clur SA (2020). Why are congenital heart defects being missed?. Ultrasound Obstet Gynecol.

[13] Bravo-Valenzuela NJ, Peixoto AB, Carrilho MC, Siqueira Pontes AL, Chagas CC, Simioni C, Araujo Júnior E (2019). Fetal cardiac function by three-dimensional ultrasound using 4D-STIC and VOCAL - an update. J Ultrason.

[14] Rizzo G, Capponi A, Cavicchioni O, Vendola M, Pietrolucci ME, Arduini D (2008). Application of automated sonography on 4-dimensional volumes of fetuses with transposition of the great arteries. J Ultrasound Med.

[15] Turan S, Turan OM, Desai A, Harman CR, Baschat AA (2014). First-trimester fetal cardiac examination using spatiotemporal image correlation, tomographic ultrasound and color Doppler imaging for the diagnosis of complex congenital heart disease in high-risk patients. Ultrasound Obstet Gynecol.

[16] Gonçalves LF, Lee W, Chaiworapongsa T (2003). Four-dimensional ultrasonography of the fetal heart with spatiotemporal image correlation. Am J Obstet Gynecol.

[17] Villafañe J, Lantin-Hermoso MR, Bhatt AB (2014). D-transposition of the great arteries: the current era of the arterial switch operation. J Am Coll Cardiol.

